# Inoperable Renal Malignant Glomus Tumor, the answers for all the “W’s”?

**DOI:** 10.15586/jkcvhl.v11i1.298

**Published:** 2024-03-01

**Authors:** Pushkala Surianarayanan, Arun Ramdas Menon, Shriley Sundersingh, Anand Raja

**Affiliations:** 1 Department of Surgical Oncology, Cancer Institute (WIA), Chennai, Tamil Nadu, India;; 2 Department of Onco-Pathology, Cancer Institute (WIA), Chennai, Tamil Nadu, India

**Keywords:** Glomus tumor, Kidney, Glomangiosarcoma, Nephrectomy, pericytic tumor

## Abstract

Glomus tumor, arising from glomus bodies (specialized neurovascular structures involved in thermoregulation), commonly occurs in extremities and rarely in viscera. The spectrum of glomus tumors range from benign tumors to tumors with uncertain malignant potential to tumors of the malignant subtype. A vast majority of visceral glomus tumors are benign. Most common visceral tumors arise in the gastrointestinal tract. Glomus tumors of the kidney are a rare entity of which malignant glomus tumors are exceedingly rare. The index patients in the existing case reports were middle-aged males. We report our experience with malignant glomus tumor of the left kidney in a 60-year-old female, with computed tomography (CT) showing involvement of renal vein and inferior vena cava (IVC). Percutaneous biopsy was performed as imaging did not conform to the appearance of a conventional renal tumor and was reported as malignant glomus tumor after immunohistochemistry. After informed decision, the patient and family elected to proceed with surgery. However, intraoperatively, the left renal mass was found to infiltrate the pancreas, duodenum, aorta, and root of the colonic mesentery due to which surgery was aborted. Biopsy obtained intraoperatively again confirmed diagnosis of left renal malignant glomus tumor. She had an uneventful postoperative recovery. Options of treatment were reviewed by a multidisciplinary board. In light of no proven benefit for systemic therapy, she was referred for supportive care. She was under follow-up and she expired after 7 months due to progressive disease. Our literature review focuses on the clinicopathologic features and the current standard of management of malignant renal glomus tumors.

## Introduction

Glomus tumors are Pericytic (perivascular) tumors of mesenchymal origin that predominantly occur in extremities and rarely involve the viscera. The family of tumors includes myopericytoma, myofibroma, angioleiomyoma, and glomus tumors and their variants. In the extremities, the cell of origin is the glomus cell, which is involved in thermoregulation. In the visceral disease, perivascular myoid cells have been hypothesized as cell of origin. The spectrum of glomus tumors ranges from benign to malignant subtypes with an intermediate group of glomus tumors with uncertain malignant potential. The histologic criteria for suspicion of high-risk glomus tumors were proposed by Folpe *et al*. (1). The theories of malignant glomus tumor’s origin have been described by Gould *et al*. (2).

The World Health Organization International Agency for Research on Cancer (WHO IARC) classification of renal tumors 2022 has brought about a complete change of definition of what malignant glomus tumors are. However, the existing criteria (1) have stood the test of time. Seventy-five percent of glomus tumors occur in extremities, 25% in viscera where the site is pre-dominantly the gastrointestinal tract. Renal glomus tumors are a rare entity and their malignant counterpart is rarer with only a few cases reported in literature. We proceed to discuss our case of malignant glomus tumor followed by the answers to all the “W” questions.

## Case Report

**Figure 1:**
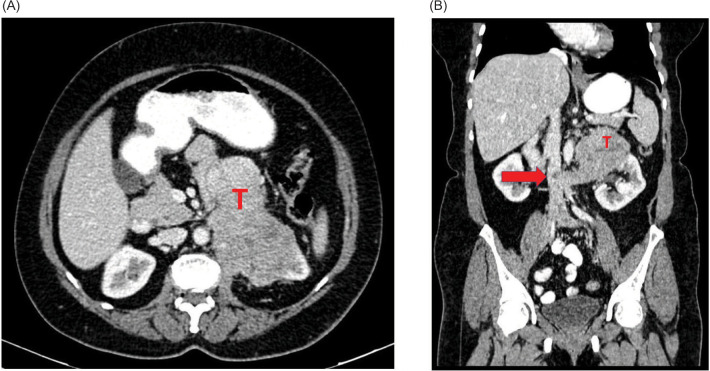
Computed tomography images showing the heterogeneously enhancing left renal mass involving the left renal vein and IVC and abutting the aorta. (A) Cross-sectional image and (B) Sagittal image. (T – Tumor), arrow pointing to the inferior vena cava (IVC) thrombus.

**Figure 2:**
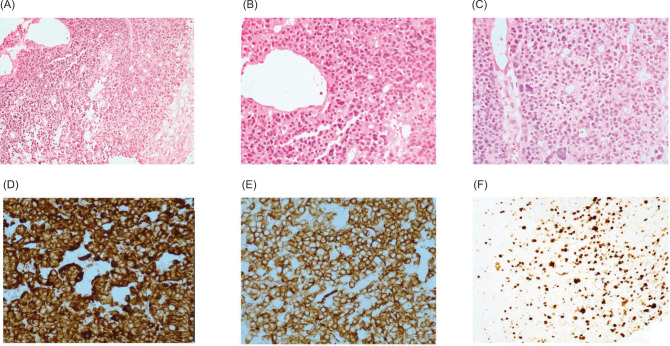
(A–C) Sheets of round cells with moderate cytoplasm and hyperchromatic nuclei with interspersed vascular spaces. H&E 200×, (D) Tumor cells express vimentin. DAB 400×, (E) Tumor cells express smooth muscle actin. DAB 400X, (F) Tumor cells show high proliferative activity with Ki67. DAB 400× DAB - Diaminobenzene.

A 60-year-old post-menopausal female presented with recent onset left flank pain. Contrast enhanced computed tomography (CECT) abdomen revealed a 11-cm partly cystic, heterogeneously enhancing left renal mass abutting the aorta with a thrombus in the left renal vein extending to the inferior vena cava (IVC) (Figure 1A,B). As the mass did not conform to the appearance of a conventional renal tumor on imaging, Image-guided percutaneous biopsy was performed. This showed fibroconnective tissue infiltrated by sheets of atypical cells showing moderate cytoplasm, hyperchromatic pleomorphic nuclei with inconspicuous nucleoli, suggesting a poorly differentiated malignant tumor (Figure 2A,C). Immunohistochemistry was positive for vimentin, smooth muscle actin (SMA), keratin, caldesmon and weakly positive for calponin, with 40% Ki 67 positivity, suggesting a diagnosis of malignant glomus tumor (Figure 2D,F). After informed decision, the patient and family elected to proceed with surgery. However, intraoperatively, the left renal mass was found to infiltrate the pancreas, duodenum, aorta, and root of the colonic mesentery due to which surgery was aborted. Biopsy obtained intraoperatively again confirmed diagnosis of left renal malignant glomus tumor. She had an uneventful post-operative recovery. Options of treatment were reviewed by the multidisciplinary board. In light of no proven benefit for systemic therapy, she was referred for supportive care. She was under follow-up and she expired after 7 months due to progressive disease.

## Discussion

### What are Glomus Tumors?

Glomus tumors are mesenchymal neoplasms belonging to the family of pericytic perivascular tumors, predominantly encountered in the extremities and less commonly in visceral organs (1). They arise from Sucquet-Hoyer canal of glomus bodies, which are specialized neurovascular organs involved in thermoregulation. The vast majority of these tumors are benign, with rare reports of malignancy (1–3).

The predominant occurrence of glomus tumors in the extremities mirrors the distribution of glomus bodies, where they grossly appear as small well-circumscribed blue–red nodules usually located in the subungual region of the nail. Twenty-five percent of glomus tumors originate in the viscera, which is peculiar in that, glomus bodies are rarely found in the viscera (4). Involvement of the gastrointestinal tract, female genital tract, bone, mediastinum, trachea, heart, lymph nodes, and the kidney have been described (4, 5).

### What makes renal glomus tumor different?

Primary renal glomus tumors are rare and have been reported to arise from the renal capsule, parenchyma, and from within the collecting system (4); however, the cell of origin is unknown. One hypothesis is that pericytic tumors are morphologically related to the differentiation of perivascular myoid cells that invest in blood vessels and function physiologically in vascular modification and thermoregulation.

Visceral glomus tumors may involve urologic organs like bladder, urethra commonly, and rarely the prostate (5, 6).

Majority of renal glomus tumors are benign. Primary malignant renal glomus tumors are exceedingly rare with only a handful of cases reported in literature (Table 1) (3). Our case is also noteworthy for the presence of an IVC thrombus.

**Table 1:** Review of literature of malignant renal glomus tumor.

Sl. No.	Author	Age/Sex	Size	Presentation	Site	Positive IHC	Management	Outcome
1	Gill (2010) (15)	46 Male	8.7 cm	Microscopic hematuria	Right kidney lower pole	SMA, MSA, vimentin, synaptophysin CD 34, BCl-2	Radical nephrectomy	NED at 15 months
2	Lamba (2010) (9)	44 Male	NA	Lower back pain due to vertebral metastasis	Right kidney, IVC thrombus	SMA, vimentin, collagen IV, CD 34	Radiation + one cycle gemcitabine Doxorubicin, dacarbazine Palliative radiation	Expired 6 months after diagnosis
3	Chen (2016) (5)	46 Male	4.5 cm	Incidental	Right kidney upper pole	NA	Radical nephrectomy	NED at 6 months
4	Lu (2016) (12)	46 Male	NA	Incidental	Right kidney upper pole	NA	Radical nephrectomy	NA
5A	Li (2018) (16)	31 Female	16 cm	Right flank pain	Right kidney	SMA, collagen IV	Right radical nephrectomy	Recurred in left kidney at 7 years, partial left nephrectomy done, recurred locoregionally after 2 years succumbs at 13 years
5B	Li (2018) (16)	33 Female	9.5 cm	Heart murmur (Tumor in the vegetation of tricuspid valve)	Left kidney	Vimentin, MSA	Radical nephrectomy, IVC thrombectomy	NA
6	Kapogiannis (2021) (11)	67 Male	2.5 cm	Incidental	Right kidney lower pole	SMA, CD57, vimentin	Partial nephrectomy	NED at 15 months
7	Nwanze (2021) (14)	32 Male	5.2 cm	Incidental	Right kidney lower pole	CD34, vimentin, SMA, caldesmon	NA	NA
8	Present case	60 Female	11 cm	Flank pain	Left kidney	SMA, Vimentin, caldesmon, calponin	Inoperable	Expired 7 months after diagnosis

SMA – Smooth Muscle Actin; MSA – Muscle Specific Actin; CD - Cluster of Differentiation; Bcl 2 – B cell lymphoma 2; NA – Not Applicable; IVC – Inferior Vena Cava; NED – No evidence of disease.

### What are the clinical characteristics of patients with renal glomus tumors?

Benign renal glomus tumors occur in the second to ninth decades of life, and in both genders (4). On the other hand, malignant renal glomus tumors have been reported in males in their 40s, with no prior reports in females. The clinical presentation is similar to those of any renal tumor, commonly an incidental diagnosis and rarely with flank pain or microscopic hematuria.

No distinctive characteristics have been described on imaging and are hence indistinguishable from other renal tumors, particularly when organ-confined. The tumor may show Standard Uptake Value (SUV) uptake in F18 Fluorodeoxyglucose (FDG)- Positron Emission Tomography (PET) due to perivascular origin (7). Consequently, diagnosis has to be made primarily on extirpative pathology (4). Our patient presented with a large tumor that did not have the appearance of a conventional renal tumor, which prompted percutaneous biopsy.

### What is the pathology of renal glomus tumors?

**Figure 3:**
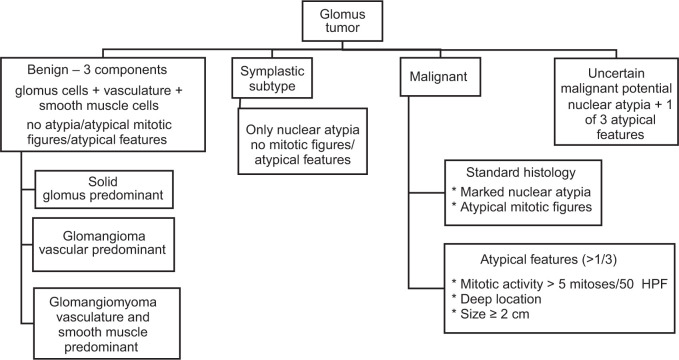
Spectrum of glomus tumor and clinicopathologic features of each subtype. HPF – high power field.

The spectrum of glomus tumors ranges from benign, tumors with uncertain malignant potential, to malignant glomus tumors, and is depicted in Figure 3. On microscopy, sheets and nests of small round cells with pale eosinophilic to amphophilic cytoplasm are seen in stroma, interspersed with vessels of varying sizes, ranging from small to large and ectatic. These tumors are classified as solid glomus tumor, glomangioma, or glomangiomyoma based on the varying predominance of neoplastic smooth muscle, vascular and stromal component (8), as described in Figure 3.

### What are malignant glomus tumors?

Goud *et al*. (2) classified aggressive/potentially malignant glomus tumors based on their histologic appearance as:

- those arising from benign glomus tumors based on the presence of benign glomus cells in periphery
- those arising de novo, based on the absence of such benign glomus cells
- locally infiltrative glomus tumors (LIGT), i.e., invasive tumors without cytologic atypia (2).

Folpe *et al*. (1) proposed that the presence of clinical and histologic features like

- deep location,
- size > 2 cm,
- atypical mitotic figures
- moderate/high-grade nuclear atypia
- ≥5 mitosis/50 high power field (HPF)

are commonly associated with malignant glomus tumors or glomus tumors with uncertain malignant potential. He has also reported the metastatic rate of malignant glomus tumors of dermal origin to be as high as 38% (1).

Our case fulfilled all criteria for suspicion of malignancy described above. Also, the presence of cytoplasmic actin and focal lattice work of collagen 4 has also been reported to indicate malignancy (9). On immunohistochemistry, unlike renal cell carcinoma, glomus tumors are negative for epithelial markers and markers native to renal origin like PAX8 (paired box gene 8)/AMACR (alpha-methylaceyl CoA racemase)/CA IX (carbonic anhydrase IX). The tumor was positive for muscle markers like Smooth Muscle Actin (SMA), vimentin, keratin and caldesmon (10) (Table 2). The marker of proliferation Ki 67 was 30–40%.

**Table 2:** Immunohistochemistry profile of our patient.

Vimentin	Positive
Smooth muscle actin (SMA)	Positive
Desmin	Negative
Muscle actin	Negative
Human melanoma black (HMB) 45	Negative
Melan A	Negative
S100P	Negative
CD 34	Negative
Inhibin	Negative
Synaptophysin	Negative
Leukocyte common antigen	Negative
Multiple myeloma oncogene 1 (MUM 1)	Negative
CD 30	Negative
CD 99	Negative
Keratin	Occasional positive
C Kit	Negative
Activin receptor-like kinase-1 (ALK1)	Negative
Calponin	Weak positive
Caldesmon	Positive

CD – Cluster of Differentiation.

P53-positive staining has been associated with malignant glomus tumors (11). B-raf proto-oncogene serine/threonine kinase (BRAF) V600E mutations have been observed in malignant renal glomus tumors, which can be utilized as a potential tumor agnostic therapy (10).

### What are familial glomus tumors?

Congenital familial multiple glomus tumors present with multifocal tumors, with incomplete penetrance, following autosomal dominant pattern of inheritance, due to inactivating mutation/uniparental disomy of Glomulin (GLMN) gene located on Chromosome 1p22.1 or biallelic inactivation of neurofibromatosis 1 (NF1) gene. NOTCH2 re-arrangements are seen in 52% of glomus tumors (10).

### What does the WHO classification of renal tumors 2022 say?

The updated WHO classification of renal tumors 2022, 5^th^ edition (10) classifies renal glomus tumors into pericytic perivascular tumors of mesenchymal origin (as was earlier). The essential diagnostic criteria are the presence of round monomorphic cells with well-defined borders with diffuse positivity for muscle markers (smooth muscle actin, muscle-specific actin, caldesmon, calponin, pericellular collagen IV), and the desirable features are negative epithelial/vascular/melanoma markers. Because of the monotonous epithelioid morphology, low-grade clear cell carcinoma can be mimicked, especially in core needle biopsy specimens.

It mentions that the ability to metastasize is the only criterion for malignant glomus tumors, notwithstanding the histologic features that distinguish benign from malignant glomus tumors that have stood the test of time.

### What are the differential diagnoses?

The differential diagnosis of primary renal glomus tumors includes renal epithelial tumors and other mesenchymal tumors. The latter include angiomyolipoma, juxtaglomerular cell tumor, hemangiomas and leiomyoma, hemangiopericytoma, leiomyosarcoma with epithelioid change, and round cell tumors, such as rhabdomyosarcoma and Ewing’s Sarcoma/Primitive Neuroectodermal tumor (PNET). This is due to the histology of uniform monomorphic round cells. Differentiation is possible by characteristic histopathologic features and immunohistochemistry. No supportive cytogenetics findings have been reported to date. The immunohistochemistry (IHC) features are described in Table 2.

### What is the treatment?

The treatment of benign glomus tumor is complete surgical excision, which, in case of glomus tumor of the kidney, involves radical nephrectomy, or partial nephrectomy, if feasible (4). Chen *et al*. hypothesized that due to the lack of a pseudo capsule or pericapsular inflammation, enucleation may lead to positive margins/recurrence (3). Despite this, excellent outcomes have been reported for benign lesions, with no recurrence (4). Majority of five out of six malignant glomus tumors reported to date have been relatively large compared to benign tumors with size ranging from 3.7 to –7.0 cm and have been managed with radical nephrectomy when organ-confined. One patient underwent partial nephrectomy (7), as the tumor size was 2.5 cm, margins were negative, and the patient was doing well at 15 months. A curative resection radical/nephron sparing with negative margins is of utmost importance in the management of these patients.

With follow-up ranging from 6 to 15 months, no recurrence has been reported in organ-confined malignant glomus tumors, suggesting that complete surgical excision is curative (5, 14, 16).

The role of active surveillance if a benign diagnosis is established unequivocally on percutaneous diagnosis is not known, although it is possible at least in theory.

Due to its rarity, limited data are available on the use of systemic therapy in locally advanced disease and metastatic disease. Prognosis is generally poor with survival ranging from 6 months to 3 years (1, 4). Radiation and gemcitabine use were associated with poor response (9). Modest response with delay in tumor growth has been reported with somatostatin and temozolomide (13).

The role of radiation is solely useful in a palliative setting to the metastatic sites like spine in cases with pain or impending malignant spinal cord compression.

Tumor agnostic therapy by use of BRAF inhibitors is yet to be studied but has a potential for opening a new horizon for therapy for patients with advanced disease.

### What is the frequency of recurrence?

Only one case has been reported to have recurred in the contralateral (left) kidney at 7 years after diagnosis with a locally infiltrative recurrence in the contralateral kidney after a nephron sparing approach at 9 years from initial diagnosis. It is noteworthy that this patient had a 16-cm right renal tumor fulfilling all criteria for a malignant glomus tumor. There is a theoretical possibility of familial multiple glomus tumor syndrome. Though the best possible chance of cure is by margin negative resection, follow-up of these patients is also important and one can extrapolate the follow-up schedule of conventional renal carcinoma to these patients until further guidelines emerge.

### What to do if there is a report of glomus tumor on histopathology?

Scenario 1 – nonmetastatic after nephron sparing procedure

Step 1 – risk stratification according to Folpe criteria (1)

Step 2 – check for margin status – absence of pseudo-capsule or peritumoral inflammation makes the tumor susceptible for positive margins in nephron-sparing approach

Step 3 – One can look for cytoplasmic actin and focal lattice work of collagen 4 which indicate malignancy in addition to Folpe criteria (as per WHO 2022, metastasis is the only indicator of malignant glomus tumor)

Step 4 – p53 and BRAF V600E testing can be desirable

Step 5 – Follow-up

Scenario 2 – advanced disease

Step 1 – curative resection offers the best possible chance for the patient

Step 2 – If inoperable, BRAF V 600E mutation can be tested for and targeted if present

Step 3 – Follow-up. Survival of 6 months to 24 months as per cases in the literature

### Where do the advanced tumor spread?

On literature review, three cases were advanced, and two had tumor thrombus in the inferior vena cava (one had tumor in the vegetation of tricuspid valve). Infiltration to adjacent structures was noted in one tumor at presentation and in one recurrent tumor. One case had distant metastases to the vertebra. None of the cases had lymph node metastasis/metastasis to the lungs.

## Conclusion

Malignant renal glomus tumor is a rare entity that is potentially curable by resection with negative margins. The pathology lays the basis of diagnosis. In advanced stages, the survival is poor with limited data for the use of conventional chemo- or radiotherapy. Tumor agnostic therapy can open up a brand new arena of scope in patients with advanced disease.

## References

[1] Folpe AL, Fanburg-Smith JC, Miettinen M, Weiss SW. Atypical and malignant glomus tumors: analysis of 52 cases, with a proposal for the reclassification of glomus tumors. Am J Surg Pathol. 2001;25(1):1–12. 10.1097/00000478-200101000-00001 11145243

[2] Gould EW, Manivel JC, Albores-Saavedra J, Monforte H. Locally infiltrative glomus tumors and glomangiosarcomas. A clinical, ultrastructural, and immunohistochemical study. Cancer. 1990;65(2):310–8. 10.1002/1097-0142(19900115)65:2<310::AID-CNCR2820650221>3.0.CO;2-Q 2153045

[3] Chen YA, Li HN, Wang RC, Hung SW, Chiu KY. Malignant Glomus Tumor of the Kidney: A Case Report and Review of the Literature. Clin Genitourin Cancer. 2017;15(1):e151–e153. 10.1016/j.clgc.2016.05.018 27349133

[4] Almaghrabi A, Almaghrabi N, Al-Maghrabi H. Glomangioma of the Kidney: A Rare Case of Glomus Tumor and Review of the Literature. Case Rep Pathol. 2017;2017:7423642. 10.1155/2017/7423642 28698815 PMC5494058

[5] Chen L, Lai B, Su X, Wang J. Unusual glomus tumor of the bladder: a rare case report and literature review. BMC Urol. 2021;21(1):66. 10.1186/s12894-021-00837-0 33882895 PMC8061168

[6] He T, Hu J, Jin LU, Li Y, Liu J, Ding YU, et al. Glomus tumor of the anterior urethra: A rare case report and review of the literature. Mol Clin Oncol. 2016;4(6):1057–59. 10.3892/mco.2016.832 27284444 PMC4887937

[7] Lu YY, Wang RC, Wang HY. Malignant glomus tumor of the kidney. Am. J. Med. Sci. 2017;353(3):310. 10.1016/j.amjms.2016.07.010 28262221

[8] Al-Ahmadie HA, Yilmaz A, Olgac S, Reuter VE. Glomus tumor of the kidney: a report of 3 cases involving renal parenchyma and review of the literature. Am J Surg Pathol. 2007;31(4):585–91. 10.1097/01.pas.0000213373.64053.41 17414106

[9] Lamba G, Rafiyath SM, Kaur H, Khan S, Singh P, Hamilton AM, et al. Malignant glomus tumor of kidney: the first reported case and review of literature. Hum Pathol. 2011;42(8):1200–3. 10.1016/j.humpath.2010.11.009 21333326

[10] WHO Classification of Tumours Editorial Board. Urinary and male genital tumours. International Agency for Research on Cancer; 2022.

[11] Hegyi L, Cormack GC, Grant JW. Histochemical investigation into the molecular mechanisms of malignant transformation in a benign glomus tumour. J Clin Pathol. 1998;51(11):872–74. 10.1136/jcp.51.11.872 10193335 PMC500988

[12] Gill J, Van Vliet C. Infiltrating glomus tumor of uncertain malignant potential arising in the kidney. Hum pathol. 2010; 41(1):145–149. 10.1016/j.humpath.2009.08.003 19896698

[13] Vasilevska-Nikodinovska V, Samardjiski M, Jovanovik R, Ilievski B, Janevska V. Low-Grade Malignancy Glomus Tumor in a Setting of Multiple Glomus Tumors-Case Report. Open Access Maced J Med Sci. 2019;7(23):4082–88. 10.3889/oamjms.2019.610 32165957 PMC7061405

[14] Kapogiannis F, Tsiampa E, Kapogiannis F. Glomus tumor of the kidney harboring malignant potential. Cureus. 2021;11:13(11). 10.7759/cureus.19479 PMC8664362 34912620

[15] Li R, Petros FG, Davis Jr CJ, Ward JF. Characterization of glomus tumors of the kidney. Clin Genitourin Cancer. 2018;16(1):e253–56. 10.1016/j.clgc.2017.09.002 28967505

[16] Nwanze J, Shih J, Rolf N, Halat SK. Malignant glomus tumor of the kidney: A case report and review of the literature. Am. J. Clin. Pathol 2021; 156: S73–S73. 10.1093/ajcp/aqab191.152

